# Towards eco‐friendly secondary plant metabolite quantitation: Ultra high performance supercritical fluid chromatography applied to common vervain (*Verbena officinalis* L.)

**DOI:** 10.1002/jssc.201900854

**Published:** 2019-12-17

**Authors:** Nora Gibitz‐Eisath, Miriam Eichberger, Regina Gruber, Christoph Seger, Sonja Sturm, Hermann Stuppner

**Affiliations:** ^1^ Institute of Pharmacy Department of Pharmacognosy CCB – Centrum of Chemistry and Biomedicine CMBI ‐ Center for Molecular Biosciences University of Innsbruck Innsbruck Austria; ^2^ Risch Laboratory Group Buchs SG Switzerland

**Keywords:** cross‐validation, orthogonality, subcritical fluid chromatography, supercritical fluid chromatography, *Verbena officinalis* L.

## Abstract

This report presents the first ultra high performance supercritical fluid chromatography diode array detector based assay for simultaneous determination of iridoid glucosides, flavonoid glucuronides, and phenylpropanoid glycosides in *Verbena officinalis* (Verbenaceae) extracts. Separation of the key metabolites was achieved in less than 7 min on an Acquity UPC^2^ Torus Diol column using a mobile phase gradient comprising subcritical carbon dioxide and methanol with 0.15% phosphoric acid. Method validation for seven selected marker compounds (hastatoside, verbenalin, apigenin‐7‐*O*‐glucuronide, luteolin‐7‐*O*‐glucuronide, apigenin‐7‐*O*‐diglucuronide, verbascoside, and luteolin‐7‐*O*‐diglucuronide) confirmed the assay to be sensitive, linear, precise, and accurate. Head‐to‐head comparison to an ultra high performance liquid chromatography comparator assay did prove the high orthogonality of the methods. Quantitative result equivalence was evaluated by Passing‐Bablok‐correlation and Bland‐Altman‐plot analysis. This cross‐validation revealed, that one of the investigated marker compound peaks was contaminated in the ultra high performance liquid chromatography assay by a structurally related congener. Taken together, it was proven that the ultra high performance supercritical fluid chromatography instrument setup with its orthogonal selectivity is a true alternative to conventional reversed phase liquid chromatography in quantitative secondary metabolite analysis. For regulatory purposes, assay cross‐validation with highly orthogonal methods seems a viable approach to avoid analyte overestimation due to coeluting, analytically indistinguishable contaminants.

**Article Related Abbreviations**
ABPR: automated back pressure regulator
CO_2_: carbon dioxide
DAD: diode array detector
MeOH: methanol
UHPSFC: ultra high performance supercritical fluid chromatography

## 1 INTRODUCTION

Modern phytoanalysis is an ever challenging application field for latest developments in analytical instrumentation technology. Secondary metabolite profiles are highly complex and feature numbers are easily exceeding peak capacities of conventional LC equipment. Hence, high resolution separation techniques are invaluable 1. Another burden phytoanalysis faces in the 21st century is the problematic use of organic solvents of petrochemical origin as methanol (MeOH) or acetonitrile due to their environmental impact. Promoting environmentally friendly “green analytical chemistry” means to reduce solvent consumption by utilizing stationary phase columns with smaller inner diameters, detectors of higher sensitivity, and HPLC instruments with lower dead volumes 2. Consequently, ultra high performance liquid chromatography (UHPLC) paired with sub‐2 µm packing columns became swiftly a key separation technology in modern phytoanalysis 3. A different approach towards eco‐friendly secondary plant metabolite quantitation is ultra high performance supercritical fluid chromatography (UHPSFC). In contrast to early SFC approaches, where pure carbon dioxide (CO_2_) in supercritical condition was used as mobile phase, modern UHPSFC applies mobile phases consisting of CO_2_ and organic solvents (mostly an alcohol). This results in mixtures that are not necessarily supercritical but rather subcritical fluids 4, 5, 6. Since characteristics of both states are comparable and boundaries seem to be artificial, we retained, as most analysts working with this technique, the term SFC in the presented publication. However, CO_2_ (regardless if in super or subcritical conditions) has major benefits over other chromatographic solvents, as it combines features of the liquid (e.g. high solvating capabilities and densities) and the gaseous state (e.g. low viscosity and high diffusivity) uniquely 7, 8. As a result, high flow rates can be applied without significant loss of chromatographic efficiency, and sub–2 µm particles columns can be used without generation of excessive pressure 9, 10, 11, 12. Consequently, UHPSFC has increasingly developed to a valuable alternative to organic solvent based reversed phase chromatography in modern natural product analysis 13, 14, 15. The main potential of UHPSFC is seen as analytical alternative for the assessment of nonpolar and moderate polar plant constituent; its applicability to more polar compounds is still less researched 13, 14, 15, 16, 17, 18, 19, 20, 21, 22. A second limitation in current literature is the focus of most validated, quantitative applications on single secondary metabolite classes. Only a few attempts have been directed towards the simultaneous quantitation of different compound classes with divergent polarity – a must in secondary metabolite profiling due to the complex structure–activity relationship in drugs derived from plant material 23, 24. Related to the complexity of secondary metabolite profiles, matrix interferences can always impair specificity of analytical methods and are thereby defined as another major challenge in natural product analysis. Even tough, the implementation of cross‐validations to compare the performance of different assays and to prove thereby the validity of obtained quantitative results is still hardly performed in phytoanalysis.

The purpose of our work was therefore on one hand to prove the applicability of UHPSFC for the simultaneous determination of three different classes of secondary metabolites with distinctive differences in polarity (log*P* values between ‐1.94 and 1.11), namely iridoid glucosides, flavonoid glucuronides, and phenylpropanoid glycosides in vervain extracts. On the other hand, we wanted to demonstrate unequivocally, that a quantitative secondary metabolite UHPSFC assay can perform comparably to a conventional UHPLC assay and that cross‐validation is a powerful tool to prove specificity. We did choose common vervain (*Verbena officinalis* L., Verbenaceae) as case study object, since this important medicinal plant has never been analyzed by SFC/UHPSFC instrumentation before. This plant has a long history of empirical medicinal use all over the world and is regulated in its use by monographs in the European, British, and Chinese Pharmacopeias. In ancient time, the topical application for the treatment of poorly healing wounds and ulcers was its main application field. Nowadays, the herbal drug *Verbenae herba* derived from *V. officinalis* is mainly used because of its expectorant, anti‐rheumatic, and diuretic effects 25, 26, 27, 28.

The experimental approach towards the novel *V. officinalis* UHPSFC method utilizing diode array detector (DAD) based signal recording included the optimization of various experimental parameters including the type of stationary phase, the composition of the organic modifier, additive concentration, column temperature, flow rate, and pressure. Subsequently, the optimized UHPSFC–DAD assay was validated according to the ICH guidelines and applied to the quantitation of the major compounds in *Verbenae herba* extracts. Finally, a head‐to‐head comparison with a recently published UHPLC–DAD 29 method was carried out in a cross‐validation experiment to demonstrate quantitative analytical equivalence of these highly orthogonal separation techniques.

## 2 MATERIALS AND METHODS

### 2.1 Chemicals and materials

All solvents and reagents (MeOH, ethanol, isopropanol, acetonitrile, phosphoric acid, trifluoroacetic acid, and formic acid) used in this study were of HPLC grade and purchased from Merck (Darmstadt, Germany). CO_2_ (4.5 grade, purity > 99.995%) was purchased from Messer (Gumpoldskirchen, Austria). Ultrapure water was produced by a Sartorius Arium 611 UV water purification system (Sartorius Stedim Biotech, Göttingen, Germany).

*Verbena officials* L. plant material batches (VO‐1–VO‐3) of Pharmacopoeia Europaea quality (*Verbenae herba*) were obtained from different pharmacies in Innsbruck, Austria. Voucher specimens are deposited at the Institute of Pharmacy, University of Innsbruck. The reference compounds hastatoside (**1**), verbenalin (**2**), apigenin‐7‐*O*‐glucuronide (**3**), cistanoside D (**4**), luteolin‐7‐*O*‐glucuronide (**5**), apigenin‐7‐*O*‐diglucuronide (**6**), verbascoside (**7**), and luteolin‐7‐*O*‐diglucuronide (**8**) were isolated and structurally characterized at the Institute of Pharmacy, University of Innsbruck 29. Purity of all reference compounds was ≥ 93% as determined by UHPLC–DAD.

### 2.2 Sample preparation

Sample preparation followed a published protocol 29. Briefly, ground *Verbenae herba* plant material (VO‐1 to VO‐3) was frozen in liquid nitrogen and homogenized with an analytical ball mill (Mikro‐Dismembrator, Sartorius, Göttingen, Germany). 100.0 ± 0.1 mg plant material was weighed into 1.5 mL polyethylene microcentrifuge tubes (Eppendorf, Hamburg, Germany), mixed with 1.0 mL ethanol/water (1:1, v/v) on a Vortex mixer (VWR, Vienna, Austria) and extracted by sonication for 10 min. After centrifugation (10621 x *g* for 5 min) supernatants were placed in a 5 mL volumetric flask. This procedure was repeated four more times, and the flask filled up to the final volume with the extraction solvent. All sample solutions were prepared in triplicated and filter prior analysis.

### 2.3 Instrumentation and analytical conditions

#### 2.3.1 UHPSFC analysis

UHPSFC‐DAD analysis was performed on an ACQUITY UPC^2^ instrument, equipped with binary solvent delivery pump, autosampler, column oven, convergence chromatography manager, automated back pressure regulator (ABPR), and a DAD (Waters, Milford, MA, USA). Optimum separation was obtained with an Acquity UPC^2^ Torus Diol 1.7 µm column (3 × 100 mm, Waters) and a mobile phase gradient formed from CO_2_ (A) and 0.15% phosphoric acid in MeOH (B). The gradient elution was programmed as follows: 87% A at 0 min, 87% A at 2.5 min, 73% A at 2.9 min, 70% A at 3.3 min, 67% A at 3.5 min, 67% A at 5.80, 64% A at 6 min and held at this composition for 1 min (total runtime: 7 min); then the column was equilibrated for 5 min under the initial conditions. Flow rate, column temperature, and ABPR were set to 1.60 mL/min, 30°C, and 130 bar. The injection volume was 1 µL, and the detection wavelengths were set to 234 and 350 nm. To minimized baseline drift, blank subtraction (MeOH injection) was performed prior to data analysis.

#### 2.3.2 UHPLC analysis

UHPLC–DAD analysis was performed according to a published protocol 29. The utilized system was an Agilent 1290 series HPLC instrument, equipped with a quaternary pump, autosampler, column oven, and a photodiode array detector (Agilent, Waldbronn, Germany). A Phenomenex Kinetex 1.7 µm XB‐C18 column (50 × 2.10 mm) guarded with an in‐line filter was used as stationary phase, and water (A) and acetonitrile (B), each fortified with 0.1% formic acid, as mobile phase solvents. The applied gradient was as follows: 95% A at 0 min, 85% A at 0.5 min, 75% A at 5 min, 65% A at 6 min, and 2% A at 6.1 min and held at this composition for 0.9 min (total runtime: 7 min); then the column was equilibrated for 5 min under the initial conditions. Flow rate, temperature, and injection volume were adjusted to 0.45 mL/min, 45°C, and 1 µL, respectively. Detection wavelengths were set to 234 and 350 nm.

### 2.4 Calibration and method validation

Validation of the UHPSFC–DAD method followed the ICH guidelines “Validation of Analytical Procedures: Text and Methodology Q2(R1)” in terms of linearity, limits of detection and quantification (LOD and LOQ), peak purity, accuracy, precision, and repeatability 30. Two stock solutions of each analyte (**1–3**, **5–8**) were prepared by separately weighing and dissolving them in ethanol/water (1:1, v/v). From these stock solutions, seven calibrator levels were prepared by serial dilution with ethanol/water (1:1, v/v). Each level was assayed in triplicate (see Table 1 for calibration data). Calibration curves were prepared by plotting the peak areas versus the concentrations of each analyte. The regression parameters (intercept, slope, and *R*²) were calculated by linear regression analysis. Estimates of the LODs and LOQs were derived from low concentration (lowest three calibrator levels) calibration function regression models as either three (LOD) or ten times (LOQ) the residual standard deviation of the y‐intercept divided by the slope 30. The lower LOQs were set to the lowest calibrator level exceeding the estimated LOQ. The upper LOQs were set to the highest calibrator level showing sufficient quantitative accuracy (bias less than 10%).

**Table 1:** UHPSFC‐DAD assay method validation results

	1	2	3	5	6	7	8
Reg. equi.	y = 620.9x–2536.1	y = 677.7x–4208.4	y = 374.4x–1027.8	y = 856.0x–1071.2	y = 511.5x–835.46	y = 588.0x–6864.2	y = 464.6x + 866.24
*R* ^2^	0.9998	0.9994	0.9997	0.9999	0.9996	0.9991	0.9987
Linearity^a^	6.4–1030.0	6.4–1075.0	6.5–921.0	5.4–869.0	5.8–897.3	9.5–953.4	6.6–1035.0
LOD^b^	1.0	0.8	1.3	1.1	0.8	1.6	2.0
LOQ^b^	3.0	2.5	3.8	3.3	2.3	4.9	6.0
Precision							
Intra‐day^c^	1.6	1.2	1.7	1.6	2.1	1.8	1.4
Inter‐day^d^	1.4	1.1	2.6	2.1	2.1	2.2	1.9
Accuracy^e^							
Low	100.2 ± 0.9	100.2 ± 2.8	−	101.7 ± 4.7	107.2 ± 2.6	96.9 ± 4.7	–
Medium	102.0 ± 2.0	100.7 ± 4.1	−	98.7 ± 2.5	105.3 ± 1.8	97.3 ± 1.9	–
High	102.5 ± 2.5	99.2 ± 1.9	−	98.1 ± 1.6	106.5 ± 2.3	104.2 ± 3.1	–

a Linearity range from LLOQ to ULOQ in µg/mL

b µg/mL

c Maximum deviation within one day based on peak area in percent (*n* = 3 on each day)

d Deviation within three days based on peak area in percent (*n* = 9)

e Recovery values (*n* = 3) in percent (mean ± RSD)

Precision was determined by triplicate analysis of three independently prepared samples (intraday precision) of VO‐1 on three consecutive days (interday precision) and expressed as the RSD of the replicate quantitative measurement of compounds **1–3,** and **5–8**. Accuracy was determined by spiking *Verbenae herba* samples (VO‐1) with different concentrations (low, medium, and high spike) of the standards **1, 2, 5, 6,** and **7** before sample workup. All samples were prepared in triplicate.

### 2.5 Calculations and statistics

Calculation of analyte concentrations and data analysis for validation was performed using Microsoft Excel 2016 (Redmond, WA). Statistical analysis of the data (regression, Passing‐Bablok regression, Bland‐Altman plots, and rank correlation analysis) was performed using MedCalc for Windows, version 18.11 (MedCalc Software, Ostend, Belgium). Linear regression analysis of the normalized retention data of all analytes was used to evaluate the degree of orthogonality between the UHPSFC and UHPLC assay. Normalized retention factors (*t*_R_^i(norm)^) were calculated according to equation: *t*_R_^i(norm)^ = (*t* ^i^_R_ – *t* ^min^_R_)/ (*t* ^max^_R_ – *t* ^min^_R_) where *t* ^max^_R_ and *t* ^min^_R_ represent the retention times of the most and least retained compounds in the data set 31. The degree of orthogonality was expressed by the *R* ^2^ of the regression analysis.

## 3 RESULTS AND DISCUSSION

### 3.1 Optimization of UHPSFC separation

**Figure 1:**
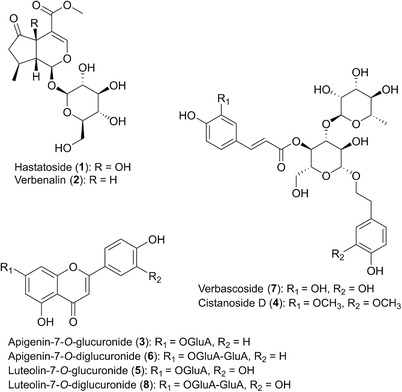
Chemical structures of the major compounds in *Verbenae herba*. Numbering in accordance with the chromatograms and tables

As the therapeutic effects of *Verbenae herba* are attributed to not only one, but three different classes of secondary metabolites, their simultaneous analysis is indispensable for adequate quality assessment. By focusing simultaneously on iridoid glucosides, flavonoid glucuronides, and phenylpropanoid glycosides by UHPSFC, one faces several problems: The analytes of interest **1–8** show distinctive differences in acid strength (p*K*_a_ values ranging from 2.60 to 12.19) as well as in polarity with log*P* values between −1.94 and 1.11. For detailed data see Supporting Information Table S1 and Figure S1. At the same time, some of the reference compounds show close structural resemblance, making a highly selective assay necessary (Figure 1). Therefore, to enable their simultaneous quantitation in the shortest possible total separation time, careful evaluation of all relevant experimental parameters including the type of stationary phase, mobile phase composition, column temperature, flow rate, and backpressure was necessary. A vervain extract (ethanol/water (1:1, v/v)) and a standard mixture of the key metabolites (**1‐8**, see Figure 1 for structures) served as samples for method development. Decisions were made mainly based on observed peak capacity, peak shape, and resolution.

Concerning the stationary phase, five different columns of the same dimensions (3 × 100 mm, 1.7 µm) were tested, including Acquity UPC^2^ HSS C 18 SB, Acquity UPC^2^ BEH, Acquity UPC^2^ CSH Fluoro Phenyl, Acquity UPC^2^ BEH 2‐EP, Acquity UPC^2^ Torus DIOL. As supercritical CO_2_ is a highly lipophilic solvent with a polarity similar to hydrocarbons, the analysis of more polar compounds requires the addition of an organic modifier 32. Therefore, MeOH (in gradient elution from 10–35%) was added for initial column screening. However, on none of the tested columns results were satisfactory: While on the C18 and the BEH column, not even signals for the more polar compounds (**3**–**8**) were observed, on the other three columns (Fluoro Phenyl, BEH 2‐EP, DIOL) their peak shapes were extremely poor. The use of other modifiers (acetonitrile, isopropanol, and mixtures thereof) did not improve the results either. Consequently, as next step, the influence of an acidic additive was investigated. For this task, phosphoric acid (0.15% v/v) was added to MeOH and the column screening with the Fluoro Phenyl, BEH 2‐EP, DIOL columns was repeated. Phosphoric acid was selected as acidic additive, as in previous studies, dealing with the SFC analysis of similar compounds, it was found to be most appropriate, explained by the minor baseline drift compared to other acidic additives at one side and its lower p*K*_a_ at the other side 33, 34. However, only on the Acquity UPC^2^ Torus DIOL column, a hybrid silica stationary phase with high‐density diol ligands, a significant improvement could be observed, resulting finally in good peak shape and satisfying resolution for all compounds. Based on these observations, the Acquity UPC^2^ Torus DIOL column was selected for all further experiments.

**Figure 2:**
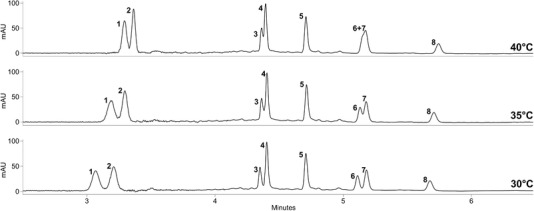
Impact of column temperature on the resolution of marker compounds **1–8**. Other parameters: column: UPC^2^ Torus Diol 1.7 µm (3 × 100 mm); mobile phase: CO_2_ (A) and 0.15% phosphoric acid in MeOH (B); gradient: 87% A at 0 min, 87% A at 2.5 min, 73% A at 2.9 min, 70% A at 3.3 min, 67% A at 3.5 min, 67% A at 5.80, 64% A at 6.0 min, held for 1 min; flow rate: 1.60 mL/min; ABPR: 130 bar; injection volume: 1 µL; detection wavelength: 234 nm

Beside the selection of an appropriated stationary phase, column temperature was another crucial factor during method development. As shown in Figure 2, a temperature of 30°C was essential for good resolution. Already an increase of 5°C resulted in co‐elution of both critical peak pairs (**3** and **4**; **6** and **7**). It is worth mentioning, that with 30°C a temperature slightly lower than the critical value (31°C) of CO_2_ was selected. Combined with the high percentage of organic modifier (up to 36%), which leads to a further increase of the critical parameters, it is obvious that the presented separation does not occur under supercritical but rather under so‐called subcritical conditions. As shown by numerous applications, using a temperature beyond the critical one is not a disadvantage as the characteristics of both states are quite comparable 4, 5, 35, 36. In SFC, a decrease of the temperature leads typically to an increase of the mobile phase density and thus to faster elution of the compounds. However, in subcritical conditions with high modifier concentrations, prediction of temperature influence is not that simple and, as reported by different studies, the influence of temperature on the retention time can change during the gradient, related to fluid compressibility changes 20, 35, 36, 37. As shown in Figure 2, this trend was observed also in the presented study. While temperature changes were affecting strongly the retention time of the early eluting compounds (**1**, **2**), the effect on the later eluting one (**3‐8**) was marginal.

**Figure 3:**
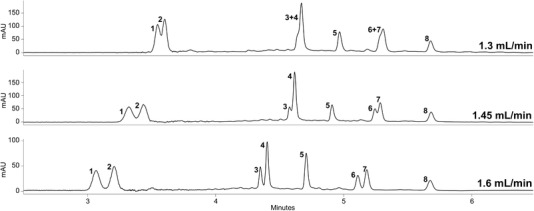
Impact of flow rate on the resolution of marker compounds **1–8**. Other parameters: column: UPC^2^ Torus Diol 1.7 µm (3 × 100 mm); mobile phase: CO_2_ (A) and 0.15% phosphoric acid in MeOH (B); gradient: 87% A at 0 min, 87% A at 2.5 min, 73% A at 2.9 min, 70% A at 3.3 min, 67% A at 3.5 min, 67% A at 5.80, 64% A at 6 min, held for 1 min; temperature: 30°C; ABPR: 130 bar; injection volume: 1 µL; detection wavelength: 234 nm

While pressure variations had only a minimal impact, flow rate changes were another key factor during method optimization. As displayed in Figure 3, the increase of the flow rate from 1.3 to 1.6 mL/min enabled not only faster elution but resulted also in better overall separation. Apigenin‐7‐*O*‐diglucuronide (**6**) and verbascoside (**7**) e.g., completely co‐eluted at a flow rate of 1.3 mL/min, while their baseline separation was observed at 1.6 mL/min. Because of the instruments pressure limit (414 bar), the effect of further flow rate increase was not investigated.

**Figure 4:**
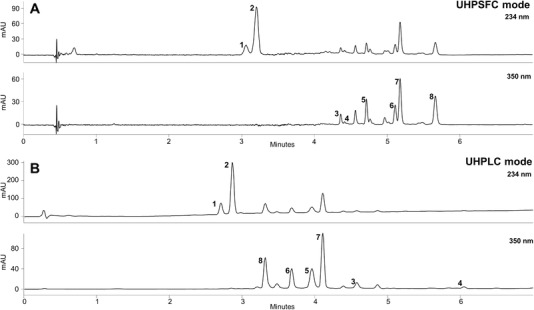
Comparison of separation by UHPSFC (**A**) and UHPLC (**B**) of a *Verbenae herba* extract under optimized conditions. Experimental conditions as described in the Section 2.3, detection wavelength 234 and 350 nm. Peak assignment in accordance with Figure 1

Finally, for optimum separation, an Acquity UPC^2^ Torus DIOL column, a temperature of 30°C, an APBR pressure of 130 bar and a flow rate of 1.6 mL/min were selected. As mobile phase, a solvent gradient of subcritical CO_2_ and MeOH containing 0.15% phosphoric acid was used. Under these optimized conditions, separation of the standard mixture (compound **1–8**) could be achieved in just seven minutes. Subsequently, all eight compounds could be assigned in the *Verbenae herba* extract (Figure 4A) by comparison of their retention times, UV‐spectra, and spiking experiments.

### 3.2 Validation of the developed UHPSFC method

The developed UHPSFC method was validated according to ICH‐guidelines for the quantitation of the major compounds **1–3**, and **5–8** in *Verbena herba*. Hence compound **4** was not available in sufficient amounts, it was not included in the validation. Depending on the chromophore type, two different wavelengths, namely 234 nm for **1** and **2,** and 350 nm for **3, 5–8,** were utilized for analyte detection.

Calibration data (Table 1) did prove assay linearity in the assigned concentration range. For all analytes, linear regression analysis *R* ^2^ did exceed 0.9987. LOD and LOQ estimates ranged from 0.8 to 2.0 µg/mL and from 2.3 to 6.0 µg/mL, respectively (Table 1). Since all LOQs were found to be below the calibration range, the LLOQ was defined to be equivalent with the lowest calibrator concentration. The overall intraday and interday variations were less than 2.6%, indicating satisfactory precision of the instrumentation and the stability of the samples (Table 1). Accuracy was determined by spiking experiments at three different concentration levels (high, medium, and low) and ranged from 96.9 to 107.2% (Table 1).

### 3.3 Sample analysis

The validated UHPSFC–DAD assay was applied to the quantitation of the seven major secondary metabolites in three different *Verbenae herba* batches. A representative UHPSFC chromatogram is displayed in Figure 4A. The obtained quantitative results (Table 2) show that each of the investigated specimens contained all investigated analytes. Verbenalin (**2**) was identified as major ingredient, followed by verbascoside (**7**) and luteolin‐7‐*O*‐diglucuronide (**8**).

**Table 2:** Quantitative UHPSFC‐DAD and UHPLC‐DAD results for compounds **1**‐**3**, **5‐8** in *Verbenae herba* samples VO‐1 ‐ VO‐3; all values expressed in mg per g plant material (with RSD in Parentheses; *n* = 3)

Compound	1	2	3	5*	6	7	8
	**UHPSFC assay**						
VO‐1	3.3 (1.4)	10.5 (1.1)	1.8 (2.6)	3.0 (2.1)	2.6 (2.1)	7.6 (2.2)	7.0 (1.9)
VO‐2	2.3 (1.0)	27.6 (0.6)	1.9 (0.9)	2.3 (1.5)	5.3 (1.0)	19.4 (1.8)	11.3 (1.8)
VO‐3	4.7 (1.4)	22.1 (0.7)	2.1 (1.8)	2.7 (2.1)	4.4 (1.1)	9.4 (1.1)	9.0 (1.8)
	**UHPLC assay**						
VO‐1	3.3 (0.7)	10.6 (0.7)	1.7 (1.1)	3.8 (2.8)	2.4 (2.2)	7.8 (1.0)	7.3 (2.8)
VO‐2	2.3 (1.3)	28.1 (0.4)	2.0 (0.4)	5.1 (0.7)	5.3 (0.6)	19.5 (1.8)	11.7 (1.3)
VO‐3	4.7 (1.8)	22.9 (1.3)	2.2 (1.2)	4.1 (1.0)	4.3 (0.4)	9.8 (1.0)	9.6 (1.1)

^*^results of the UHPLC assay are very likely overestimated due to a possible co‐elution

### 3.4 Comparison of UHPSFC and UHPLC method

In a previous study, presented by our institution, a UHPLC–DAD method for the quantitative quality control of *Verbena herba* has been developed 29. The optimized conditions are described in Section 2.3.2. As shown by the chromatograms displayed in Figure 4, both assays enable a rapid separation of the major compounds within 7 min. The elution order of flavonoid glucuronides and phenylpropanoid glycosides in LC mode is entirely different from that in SFC mode. To determine the degree of orthogonality, normalized retention times of one method were plotted against that of the other method (see Supporting Information Figure S2) and the correlation calculated by linear regression analysis. The high degree of scatter and the low *R* ^2^ value (*R* ^2^ = 0.0914) are indicating the high level of orthogonality between both assays 38. This can be explained by different retention mechanisms at one side and by different mobile (UHPSFC: CO_2_ and MeOH; UHPLC: H_2_O and ACN) and stationary phase (UHPSFC: DIOL; UHPLC: C18) characteristics at the other side. In RP–HPLC, due to the hydrophobic stationary phase, hydrophobic interactions are mainly determining retentivity. In contrast, hydrogen bonding and ionic interactions‐based separation mechanisms are dominating in UHPSFC if polar, high‐density DIOL columns, are used 10.

In terms of validation, for the investigated compoounds (**1–3**, **5–8**) both techniques showed satisfying and comparable results for the recommended criteria such as linearity range, (UHPSFC: *R* ^2^ ≥ 0.9987; UHPLC: *R* ^2^ ≥ 0.9990), and accuracy (recovery rates between 93.9 and 108.8% for UHPLC, and 96.9 to 107.2% for UHPSFC). With LOQ values between 2.3 and 6.0 µg/mL for the UHPSFC method and values between 1.1 and 7.0 µg/mL for the UHPLC method, also in terms of sensitivity, a large degree of conformity could be observed.

With a solvent consumption of 2.3 mL MeOH per run for the UHPSFC assay and 1.0 mL ACN for the UHPLC assay, both assays are presenting an eco‐friendly strategy for fast secondary plant metabolite quantitation. The higher solvent consumption in the UHPSFC assay is maybe surprising for most readers, especially as the technique has often been praised for its green character compared to organic solvent based RP‐chromatography. However, first one has to consider that the presented work compares an UHPSFC assay with a modern, state‐of‐the‐art UHPLC assay and not, like most published comparisons, with a conventional HPLC assay. Second, it focuses on polar compounds with up to two sugar residues and therefore, the necessity of a gradient with high organic modifier concentration in UHPSFC mode is only logical.

**Figure 5:**
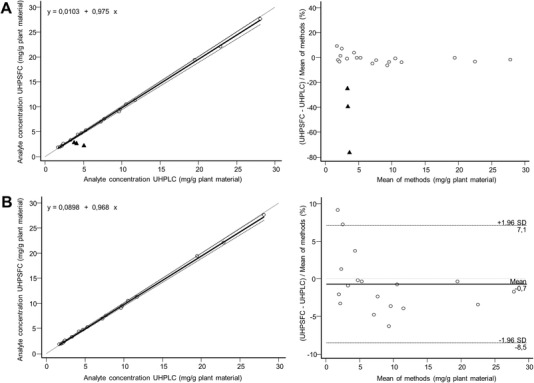
Passing‐Bablok correlation (left‐hand column) and relative Bland‐Altman plot (right‐hand column) analysis for comparison of the quantitative results of UHPLC‐DAD and UHPSFC‐DAD measurements of *Verbenae herba* samples. A: all analytes are included; luteolin‐7‐*O*‐glucuronide (**5**) is displayed as black triangle, all other analytes as open circles. B: Luteolin‐7‐*O*‐glucuronide (**5**) was excluded

To compare the quantitative performance, extracts of three different *Verbena herba* batches (VO‐1–VO‐3) were prepared in triplicate and used for UHPSFC as well as for UHPLC analysis. Comparison of the results (Table 2) did prove that both techniques are equivalent regarding the quantitative assessment of compounds **1–3** and **6**–**8**. For example, the content of verbenalin (**2**), the quality determining parameter according to the European Pharmacopeia, differed only by 0.08 mg per g plant material (0.8%) in sample VO‐1. With a deviation of 0.18 mg per g plant material (2.4%), a similar trend was observed for verbascoside (**7**). Luteolin‐7‐*O*‐glucuronide (**5**) however, showed a significant method bias: Whilst an amount of 3.77 mg/g plant material was determined by the UHPLC assay, only 2.95 mg per g were detected in the UHPSFC assay, resulting in an absolute bias of 24.6%. Consequently, in a Bland‐Altman‐plot based method comparison analysis of all individual analysis results as well as in the corresponding Passing‐Bablok correlation (Figure 5A), a significant bias was observed for this compound in all samples.

The most plausible explanation for this effect is that an unidentified congener is coeluting with luteolin‐7‐*O*‐glucuronide (**5**). Obviously, this substance was not detectable in the UHPLC–DAD–MS setting 29. Hence its UV spectrum and the ions formed under the selected MS conditions are very similar to **5**. One likely hypothesis which is supported by this experimental finding is, that this secondary metabolite is an isobaric geometrical isomer of luteolin‐7‐*O*‐glucuronide with very similar spectroscopic properties and gas phase reaction products under ionizing conditions; e.g., luteolin‐5‐*O*‐glucuronide. This hypothesis is supported by the batch to batch http://inconstancy of the bias, the overestimation by UHPLC is: 24.6% for VO‐1, 75.7% for VO‐2, and 38.9% for VO‐3. Due to the orthogonal character of UHPSFC to UHPLC, the novel assay provided a different elution order (Figure 4), which seems to be advantageous in this specific case.

After exclusion of luteolin‐7‐*O*‐glucuronide (**5**) from data comparison, Passing‐Bablok correlation analysis of UHPLC and UHPSFC derived analyte contents in samples VO‐1‐VO‐3 did result in ideal correlation equations (Figure 5B, lefthand‐column) with intercepts statistically not different from zero (intercept = 0.089, 95% confident interval = ‐0.001 to 0.251) and slopes statistically hardly different from one (slope = 0.968, 95% confident interval = 0.945 to 0.987) 39. The spearman rank *R* ^2^ was found to be 1.000 confirming the visually impressive correlation. The results of the Passing‐Bablok correlation analysis were further confirmed by relative Bland‐Altman‐plot based method comparison analysis (Figure 5B, right‐hand column) which showed a mean analysis result bias of −0.7% 40. The 2SD confidence interval (2SD = 7.8%) was in good agreement with the interday RSD data reported in the validation process of the assay and the RSD data obtained from repeated analysis of the samples (Table 1).

## 4 CONCLUDING REMARKS

Taken together, the results are proving the excellent suitability of UHPSFC for the separation and quantitative analysis of polar secondary metabolite structure classes as iridoid glucosides, flavonoid glucuronides, and phenylpropanoid glycosides. For the chosen case study plant, *V. officinalis*, UHPSFC based analysis of *Verbena herba* extracts was found to be equivalent to UHPLC based analysis in terms of analysis speed, sensitivity and selectivity for most compounds. Evaluation of the method accuracy of the UHPSFC method with a cross‐validation experiment against a UHPLC assay unveiled, that for one out of seven analytes a hitherto undiscovered congener coelution was detectable. The UHPSFC resolution of this peak pair coeluting in the UHPLC assay does highlight the importance of method cross‐validation in natural product analysis, where matrix interferences related to the complexity of the samples are one of the major challenges and can always impair the specificity of analytical methods.

## CONFLICT OF INTEREST

The authors have declared no conflict of interest.

## Supporting information

Supporting information
