# Supplementary material for: Towards eco‐friendly secondary plant metabolite quantitation: Ultra high performance supercritical fluid chromatography applied to common vervain (Verbena officinalis L.)
Source: J Sep Sci. 2019 Dec 17;43(4):829–38. doi: 10.1002/jssc.201900854 (PMC7160600; doi:10.1002/jssc.201900854)
Supplement: Supplementary file 1 — Supporting information [file JSSC-43-829-s001.docx]

**SUPPLEMENTARY MATERIALS**

**Towards eco-friendly secondary plant metabolite quantitation: ultra-high performance supercritical fluid chromatography applied to common vervain (*Verbena officinalis* L.)**

Nora Gibitz-Eisath^1^, Miriam Eichberger^1^, Regina Gruber^1^, Christoph Seger^1,2^, Sonja Sturm^1^, Hermann Stuppner^1^

**Affiliation**

^1^Institute of Pharmacy, Department of Pharmacognosy, CCB – Centrum of Chemistry and Biomedicine, CMBI - Center for Molecular Biosciences, University of Innsbruck, Innsbruck, Austria

^2^Risch Laboratory Group, Buchs, SG, Switzerland

**Correspondence**

Dr. Sonja Sturm, Institute of Pharmacy, CCB – Centrum of Chemistry and Biomedicine, University of Innsbruck, Department of Pharmacognosy, Innrain 80/82, 6020 Innsbruck, Austria. Phone: +4351250758408 Fax: +4351250758499 e-mail: [sonja.sturm@uibk.ac.at](mailto:sonja.sturm@uibk.ac.at)

**Supplementary Table S1.** Predicted physicochemical properties of reference compounds **1-8**

|  | Name | Compound class | H-bond acceptors* | H-bond donor* | logD  pH 5* | logP* | Strongest acidic pKa* |
| --- | --- | --- | --- | --- | --- | --- | --- |
| 1 | **Hastatoside** | iridoid glucoside | 10 | 5 | -1.94 | -1.94 | 11.65 |
| 2 | **Verbenalin** | iridoid glucoside | 9 | 4 | -1.45 | -1.45 | 12.19 |
| 3 | apigenin-7-*O*-glucuronide | flavonoid glucuronide | 11 | 6 | -1.48 | 0.76 | 2.74 |
| 4 | cistanoside D | phenylpropanoid glycosides | 14 | 7 | 1.11 | 1.11 | 9.69 |
| 5 | luteolin-7-*O*-glucuronide | flavonoid glucuronide | 12 | 7 | -1.79 | 0.46 | 2.74 |
| 6 | apigenin-7-*O*-diglucuronide | flavonoid glucuronide | 17 | 9 | -4.85 | -0.69 | 2.60 |
| 7 | verbascoside | phenylpropanoid glycosides | 14 | 9 | -0.82 | 0.82 | 9.01 |
| 8 | luteolin-7-*O*-diglucuronide | flavonoid glucuronide | 18 | 10 | -5.15 | -1.00 | 2.60 |

*Data obtained from ChemAxon Software (<https://chemicalize.com>; accessed on 2019-07-15)

**Figure S1.** Predicted physicochemical properties of reference compounds **1-8**. Each compound was plotted according to its predicted lipophilicity and ionization constant (logP and pK_a_ values). Iridoid glucosides (blue squares triangles), flavonoid glucuronides (green triangles), and phenylpropanoid glycosides (red dots). Numbering in accordance with figure 1.


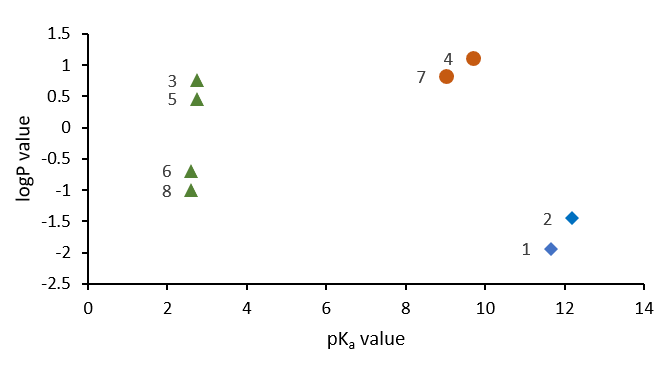


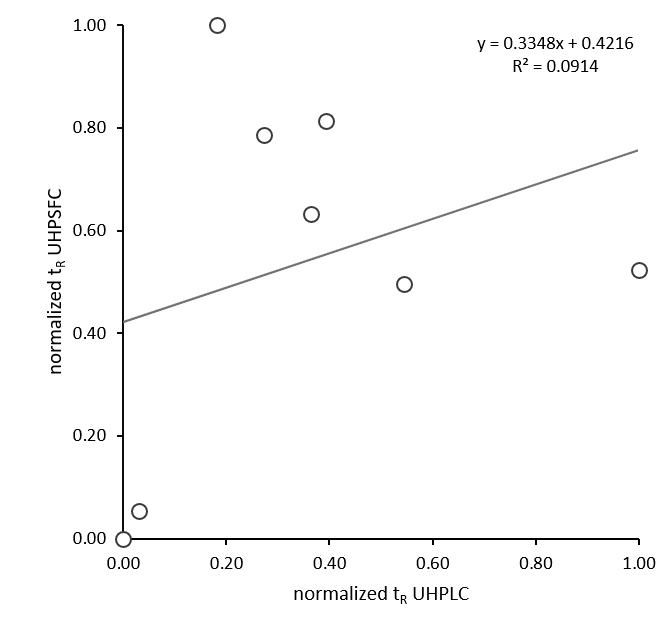
**Figure S2.** Comparison of normalized retention times of reference compounds **1-8** obtained with the UHPSFC and the UHPLC assay.
